# Cardiac cell type-specific responses to injury and contributions to heart regeneration

**DOI:** 10.1186/s13619-020-00065-1

**Published:** 2021-02-02

**Authors:** Weijia Zhang, Jinxiu Liang, Peidong Han

**Affiliations:** Division of Medical Genetics and Genomics, Children’s Hospital, Zhejiang University School of Medicine and National Clinical Research Center for Child Health, Hangzhou, China

**Keywords:** Heart regeneration, Cardiomyocytes, Endocardial cells, Epicardial cells, Fibroblasts, Immune cells

## Abstract

Heart disease is the leading cause of mortality worldwide. Due to the limited proliferation rate of mature cardiomyocytes, adult mammalian hearts are unable to regenerate damaged cardiac muscle following injury. Instead, injured area is replaced by fibrotic scar tissue, which may lead to irreversible cardiac remodeling and organ failure. In contrast, adult zebrafish and neonatal mammalian possess the capacity for heart regeneration and have been widely used as experimental models. Recent studies have shown that multiple types of cells within the heart can respond to injury with the activation of distinct signaling pathways. Determining the specific contributions of each cell type is essential for our understanding of the regeneration network organization throughout the heart. In this review, we provide an overview of the distinct functions and coordinated cell behaviors of several major cell types including cardiomyocytes, endocardial cells, epicardial cells, fibroblasts, and immune cells. The topic focuses on their specific responses and cellular plasticity after injury, and potential therapeutic applications.

## Background

Coronary artery occlusion induced cardiac infarction leads to extensive cell death. Although a small portion of cardiomyocytes in the wound border display cell cycle activities, cell division events are rare and insufficient to restore damaged myocardium. Instead, injured area is replaced by fast dividing fibroblasts, which give rise to fibrotic scar tissue and further promote cardiac structural remodeling and deteriorated function. Thus, one major goal of cardiac regenerative medicine is to develop therapeutic strategies that boost the intrinsic proliferation of cardiomyocytes for functional recovery. To this end, various animals have been used as in vivo models to explore heart regeneration. Initial studies were conducted in lower vertebrates such as newts (Oberpriller & Oberpriller, 1974) and axolotls (Flink, 2002), as they have displayed a broad range of regenerative capacities in multiple organs. Experimental results indicated the presence of cardiomyocyte proliferation that partially replaced injured myocardium. However, due to a lack of genetic tools to dissect the underlying molecular mechanisms, progress in these animal models has been relatively slow.

In 2002, adult zebrafish heart regeneration was first reported (Poss et al., 2002). Compared with newt and axolotl, a major advantage of the zebrafish model is the availability of a large number of transgenic or gene deletion strains, which are essential to define the function of specific genes. As a result, key molecular and cellular events underlying zebrafish heart regeneration have been discovered in the past two decades, such as the activation of epicardium (Kikuchi et al., 2011a; Lepilina et al., 2006), changes in epigenetic programming (Xiao et al., 2016), reactivation of key cardiac developmental related transcription factor genes (Kikuchi et al., 2010), and disassembly of cardiomyocyte sarcomeric structure (details of these events will be discussed in the following sections). However, hearts in lower vertebrates are generally considered immature when compared with those of adult mammalians due to their highly trabeculated cardiac structure and a lack of transverse tubules in cardiomyocytes. Thus, there remains a need for a cardiac regeneration model in the mammalian. In 2011, a breakthrough was achieved in the discovery of regeneration in a neonatal mouse model (Porrello et al., 2011). As opposed to the persistent regenerative capacity exhibited throughout life in zebrafish, this time window stops at postnatal day 7 in mouse models. Nevertheless, advanced genetic tools in mammalians such as conditional knockout and knockin models have provided the opportunity to acquire additional information regarding molecular and cellular mechanisms involved. Overall, our current knowledge of heart regeneration comes from studies in adult lower vertebrate such as zebrafish (Poss et al., 2002), newt (Oberpriller & Oberpriller, 1974) and axolotl (Flink, 2002), as well as neonatal mammalian including mouse (Porrello et al., 2011), rat (Wang et al., 2020), pig (Zhu et al., 2018; Ye et al., 2018), and human (Haubner et al., 2016).

**Fig. 1:**
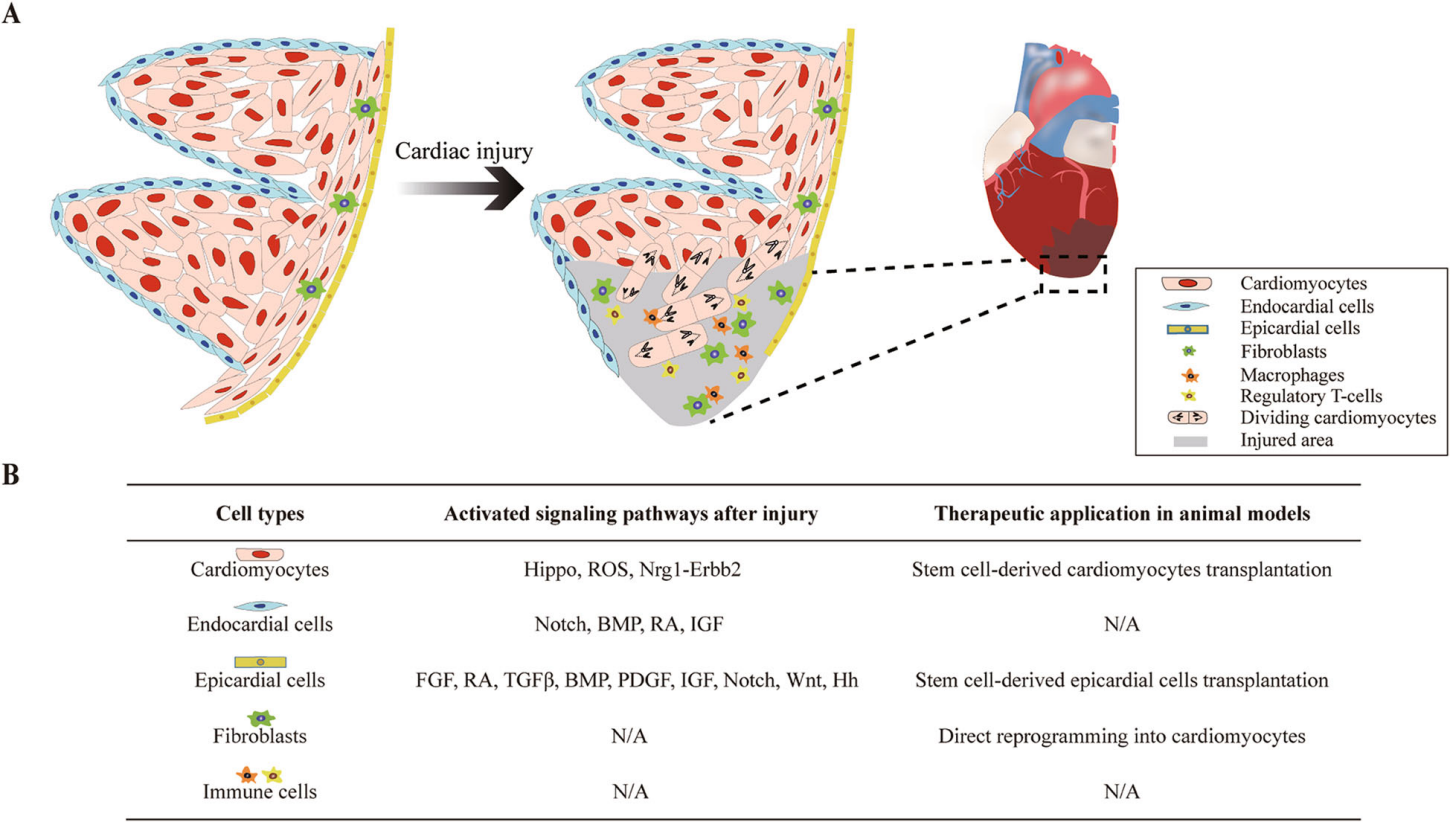
Cell type-specific responses to cardiac injury. **a** The outer surface and luminal surface of the myocardium is covered by epicardial cells and endocardial cells, respectively. Fibroblasts reside between cardiomyocytes within the myocardium. After injury, the activation of epicardial cell, endocardial cells, fibroblasts, and immune cells, together with proliferation of cardiomyocytes, contribute to heart regeneration. **b** Summary of activated signaling pathways after injury and applications in animal models

Adult heart contains multiple cell types including cardiomyocytes, endocardial cells, epicardial cells, fibroblasts, immune cells, blood cells, and vascular smooth muscle cells, which play distinct physiological roles. The major function of cardiomyocytes is to generate rhythmic contractions that maintain circulation. Endocardial cells form a lining within the cardiac lumen as a physical barrier for blood flow. On the outer surface of the heart, a thin layer of epicardial cells covers the entire myocardium that can give rise to coronary vascular smooth muscle cells and fibroblasts. In addition, with interspersed distributions between cardiomyocytes, there are abundant fibroblasts which synthesize extracellular matrix (ECM) to provide mechanical support for the heart. Overall, these different cell types establish cell-cell communications via direct contact or through secreted signaling molecules to maintain physiological cardiac function in a resting state. Following injury, the proliferation of a subpopulation of cardiomyocytes is reliant on extrinsic cues from epicardial, endocardial cells, fibroblasts, and immune cells. This tightly coordinated behavior of various cell types is required for morphological and functional regeneration (Fig. 1).

## Main Text

### Cell type-specific responses

#### Cardiomyocytes

Cardiomyocytes are elementary units of contractile function within the heart. Each heartbeat starts with an electrical impulse from pacemaker cells, which propagates through the cardiac conduction system and triggers synchronized contraction of cardiomyocytes in order to drive the circulation of blood flow. The mammalian heart develops from two distinct cardiac progenitor populations: the first heart field, which gives rise to the left ventricle and majority of the atria; and the second heart field, which contributes to the right ventricle, parts of the atria and outflow tract. Cardiac progenitor cells in both fields further give rise to two layer of cells in the developing heart tube consisting of an inner layer of endocardial cells and an outer layer of cardiomyocytes (Chien et al., 2008).

Under a resting state, mature mammalian cardiomyocytes exhibit a very limited turnover rate estimated at less than 1% per year in adult human hearts (Bergmann et al., 2009; Bergmann et al., 2015). After injury, although part of the cardiomyocytes close to wound border display cell cycle activities, authentic cell divisions are extremely low and insufficient to restore lost cells. In contrast, adult lower vertebrate or neonatal mammalian cardiomyocytes present a strong proliferative capacity and hearts are able to regenerate after injury. Genetic fate mapping studies have demonstrated that regenerated heart muscle is derived from preexisting cardiomyocytes (Kikuchi et al., 2010; Porrello et al., 2011; Jopling et al., 2010), while contributions from resident stem or progenitor cells is minimal (Li et al., 2018). Furthermore, regenerating cardiomyocytes undergo a limited extent of reprogramming, characterized by reactivation of the essential cardiac developmental related transcription factors such as *gata4 (* Kikuchi et al., 2010*)* and *hand2 (* Schindler et al., 2014*)*, adoption of a more glycolytic metabolism state (Honkoop et al., 2019), and disassembly of sarcomeric ultrastructure (Jopling et al., 2010; Engel et al., 2005; Engel et al., 2006; Ahuja et al., 2004). Interestingly, adult mammalian cardiomyocytes exhibit disassembled centrosome which is associated with cell cycle arrest, whereas centrosome integrity in adult lower vertebrates or neonatal mammalian cardiomyocytes remains intact (Zebrowski et al., 2015). In addition, heterochromatin accumulation and targeting of proliferation-activating genes to the transcriptionally silent regions has also been shown as the cell cycle exit mechanism in adult mammalian cardiomyocytes (Sdek et al., 2011). Nevertheless, how the intrinsic differences between regenerating and non-regenerating cardiomyocytes lead to divergent responses to injury remains elusive.

Our current knowledge of the activated signaling pathways within regenerating cardiomyocytes includes: (1) **Hippo signaling**: The mammalian Hippo signaling core components include Mst1 and Mst2, which form a complex with Salvador (Salv) to phosphorylate Lats1 and Lats2. Lats1/2 kinases further phosphorylate transcriptional co-activators Yap and Taz to exclude them from cell nuclei and limit their transcriptional activity. Salv gene knockout study has demonstrated that Hippo signaling limits cardiomyocyte proliferation and heart size during development (Heallen et al., 2011). After myocardial infarction, Hippo signaling deficient adult hearts display efficient regeneration with reduced scar size (Heallen et al., 2013; Leach et al., 2017). In addition, cardiomyocyte-specific expression of a constitutively active Yap also enhances regeneration and contractile function after infarction (Xin et al., 2013). Recent studies further demonstrate that Hippo signaling is regulated by cardiac tissue stiffness and ECM rigidity. The dystrophin glycoprotein complex (DGC), which links cardiomyocyte actin cytoskeleton structure to ECM, directly binds to Yap and inhibits cell proliferation (Morikawa et al., 2017). Moreover, ECM glycoprotein agrin stimulates the proliferation of cardiomyocytes through the disassembly of DGC and subsequent Yap translocation into the nuclei (Bassat et al., 2017). (2) **Reactive oxygen species (ROS) signaling**: The major sources of ROS production are NADPH oxidase (Nox), dual oxidase (Duox) on the cell membrane, and mitochondria. Compared with adult mammalian heart, embryonic heart resides in an environment of low oxygen level. Shortly after birth, the increased oxygenation state and mitochondrial content, and the shift from glycolysis to oxidative metabolism leads to elevated ROS production in cardiomyocytes, which triggers DNA damage response and cardiomyocytes cell cycle arrest, as well as loss of regenerative capacity (Puente et al., 2014). Likewise, it has also been reported that Pitx2 promotes neonatal heart regeneration by activating ROS scavengers for its clearance (Tao et al., 2016). Accordingly, when adult mice are gradually exposed to systemic hypoxia, decreased ROS and DNA damage is sufficient to induce a robust regenerative response after infarction (Nakada et al., 2017). In zebrafish, cardiac resection induces epicardial *nox/duox* expression and ROS component H_2_O_2_ production. Elevated ROS destabilizes the redox sensitive phosphatase *dusp6*, a key negative regulator of *erk1/2*, thus activates MAP kinase signaling to promote myocardial regeneration (Han et al., 2014). (3) **Nrg1-Erbb2 signaling**: The function of this signaling pathway has been studied extensively during mammalian (Gassmann et al., 1995; Lee et al., 1995; Meyer & Birchmeier, 1995) and zebrafish cardiac trabeculation (Liu et al., 2010; Han et al., 2016). Endocardial cell secreted Neuregulin 1 (Nrg1) binds to its receptor Erbb2/Erbb4 heterodimer on cardiomyocytes membrane and triggers downstream signaling cascade to promote cell proliferation and migration. After adult zebrafish heart injury, expression of *nrg1* is elevated in perivascular cells, while inhibition of this signaling pathway suppresses regeneration (Gemberling et al., 2015). Similarly, in neonatal mice hearts, transient Erbb2 activation extends the regenerative window beyond the first week of postnatal life (D'Uva et al., 2015).

Although fine-tuning of these signaling pathways is effective in animal models to reactivate the intrinsic proliferative capacity of cardiomyocytes, applying such knowledge in a spatiotemporal and tissue-specific manner for therapeutic purposes still remains technically challenging. A recent review has critically analyzed the current literature regarding strategies to induce cardiomyocyte proliferation and heart regeneration (Leone & Engel, 2019). In addition, an alternative approach to rejuvenate injured heart is utilizing embryonic stem cells (Shiba et al., 2012; Chong et al., 2014; Liu et al., 2018) or induced pluripotent stem cells (Shiba et al., 2016; Liang et al., 2019). When injected into injured myocardium, these stem cell-derived cardiomyocytes partially engraft into the heart and provide functional improvement. However, a subset of animals also experience ventricular arrhythmias due to graft-associated ectopic pacemaker activities, suggesting incomplete electrophysiological coupling between implanted and host cardiomyocytes (Liu et al., 2018). Interestingly, a recent study argues the cardiac function enhancement after stem cell therapy is not associated with de novo cardiomyocyte production. Instead, an acute inflammatory based wound healing response, mediated by macrophages, is essential for the restoration of the mechanical properties of injured heart (Vagnozzi et al., 2020).

#### Endocardial cells

Endocardial cells are a thin layer of specialized endothelial cells which cover the luminal surface of the heart that provides a physiological barrier for blood circulation. During development, endocardial cells can further give rise to cushion mesenchyme cells through endothelial-to-mesenchymal transition to form heart valve (de Lange et al., 2004; Lincoln et al., 2004). They also contribute to cardiac pericytes, smooth muscle cells (Chen et al., 2016), and adipocytes (Zhang et al., 2016) through intermediate mesenchymal stages. In addition, a subpopulation of the endocardial cells bud out from the heart lumen and directly generate part of the coronary endothelial cells (Red-Horse et al., 2010; Wu et al., 2012; Tian et al., 2014). Furthermore, endocardial cells promote cardiac trabeculation and cardiomyocyte maturation through paracrine signaling.

When cardiac tissue homeostasis is interrupted by injury, genetic fate mapping studies demonstrate that endocardial cells minimally contribute to coronary endothelial cells (Tang et al., 2018). Instead, they serve as an important signaling center for heart regeneration. The activated signaling pathways include: (1) **Notch signaling**: Activation of Notch signaling has been studied in a variety of injury models. Following zebrafish heart resection injury, expression levels of *notch1a*, *notch1b,* and *notch2* are prominently elevated in the endocardium (Zhao et al., 2014). Likewise, after cryoinjury, *notch1b*, *notch2,* and *notch3* are induced in endocardial cells (Munch et al., 2017). In addition, by using a ventricular cardiomyocytes-specific ablation system together with a Notch reporter transgenic line, increased Notch activity has been observed in atrial endocardial cells after embryonic heart injury, which results from elevated *notch1b* and *deltaD* expression (Zhang et al., 2013; Galvez-Santisteban et al., 2019). Pharmacological or genetic suppression of Notch activity consistently inhibits cardiomyocyte proliferation and impairs heart regeneration in these studies (Zhao et al., 2014; Munch et al., 2017; Zhang et al., 2013; Galvez-Santisteban et al., 2019; Zhao et al., 2019; Raya et al., 2003). However, it should be noted that hyperactivation of Notch signaling has controversial effects on cardiomyocyte proliferation (Zhao et al., 2014; Munch et al., 2017). The reason for this controversy is still unclear. (2) **Bone Morphogenetic Protein (BMP) signaling**: Phosphorylated Smad1/5/8, which reflects the activation of Bmp signaling, is detected in endocardial cells, epicardial cells, and cardiomyocytes in injured zebrafish heart (Wu et al., 2016). Additionally, expression of BMP ligands *bmp2b*, *bmp7*, together with the receptor *bmpr1aa*, are elevated in cells surrounding the injury zone, albeit the identities of these cells remain to be elucidated. Overexpression of Bmp antagonist *noggin3* impairs regeneration, whereas heart overexpressing Bmp ligand *bmp2b* exhibits an opposite effect. However, the exact role for BMP signaling in endocardial cells still remains elusive, since phosphorylated Smad1/5/8 presents in multiple cell types, and the aforementioned genetic studies are not conducted in a tissue-specific manner. (3) **Retinoic Acid (RA) signaling**: Expression of the RA synthesizing enzyme *raldh2* is rapidly induced in endocardial and epicardial cells after zebrafish (Lepilina et al., 2006; Kikuchi et al., 2011b) or mouse (Porrello et al., 2011) heart injury. Overexpression of the RA-degrading enzyme *cyp26a1* or the dominant negative RA receptor leads to inhibition of this signaling pathway and defective cardiac regeneration (Kikuchi et al., 2011b). Nevertheless, cell type-specific techniques are still required to define the effects of RA signaling in the endocardium. (4) **Insulin-like Growth Factor (IGF) signaling**: In the regenerating zebrafish heart, the ligand gene *igf2b* is detected in endocardial cells and epicardial cells (Choi et al., 2013; Huang et al., 2013), whereas its receptor *igfr1* expresses in cardiomyocytes (Choi et al., 2013). Blocking IGF signaling decreases cardiomyocyte proliferation and impairs heart regeneration (Choi et al., 2013; Huang et al., 2013), while treatment with an IGF signaling agonist results in an opposite effect (Choi et al., 2013).

#### Epicardial cells

Epicardial cells originate from transient embryonic extracardiac tissue, namely the proepicardium. During development, proepicardial cells protrude, attach to, and finally cover the surface of embryonic myocardium to form a layer of epicardium (Maya-Ramos et al., 2013). Cellular identity of epicardial cells is defined by expression of Transcription factor 21 (Tcf21), T-box 18 (Tbx18) or Wilms’ tumor 1 (Wt1) (Acharya et al., 2012; Zhou et al., 2008; Cai et al., 2008). Epicardial cells can further give rise to cardiac fibroblasts and coronary vascular smooth muscle cells through epithelial-to-mesenchymal transition and provide structural support for the heart (Zhou et al., 2008; Cai et al., 2008).

After resection injury in both zebrafish and neonatal mice, re-expression of Raldh2 (Lepilina et al., 2006; Kikuchi et al., 2011b), Tbx18 (Schnabel et al., 2011), or Wt1 (Porrello et al., 2011; Schnabel et al., 2011; Gonzalez-Rosa et al., 2011), and rapid epicardial activation have been reported. Studies in the past two decades have demonstrated the involvement of multiple signaling pathways in epicardial cells, including Fibroblast Growth Factor (FGF), RA, Transforming Growth Factor-beta (TGFβ) and BMP, Platelet-Derived Growth Factor (PDGF), IGF, Notch, Wnt/β-catenin, and Hedgehog (Hh) signaling. The distinct roles of these signaling pathways have been reviewed elsewhere (Cao & Poss, 2018). In general, activation of these signaling pathways either regulates the proliferation of epicardial cells or mediates crosstalk between epicardial cells and other cell lineages.

In addition, studies in model organisms have demonstrated that the contribution of epicardial cells to cardiomyocytes is minimal after injury (Kikuchi et al., 2011a; Christoffels et al., 2009; Rudat & Kispert, 2012). Instead, they transdifferentiate into vascular smooth muscle cells or pericytes (Kikuchi et al., 2011a; van Wijk et al., 2012; Zhou et al., 2011), which are essential for cardiac repair and scar formation. In order to utilize such characteristics of epicardial cells for therapeutic purposes, different strategies have been applied to generate epicardial cells from pluripotent stem cells in vitro (Witty et al., 2014; Iyer et al., 2016). These epicardial cells express markers like Tbx18, Wt1 and Aldh1a2 and are able to transdifferentiate into fibroblasts and vascular smooth muscle lineages when epithelial-to-mesenchymal condition is induced, corroborating the epithelial identity. Accordingly, when transplanted into infarcted heart, these stem cell-derived epicardial cells transdifferentiate into fibroblasts (Bargehr et al., 2019). Furthermore, co-transplantation of epicardial cells and cardiomyocytes results in larger graft size, increased host vascularization, and improved systolic function (Bargehr et al., 2019), making them a promising therapeutic target for heart regeneration.

#### Fibroblasts

The major developmental source of mammalian cardiac fibroblast is the embryonic epicardium, while the endocardium also contributes to a relatively small portion (Moore-Morris et al., 2014). Fibroblasts are essential for maintaining normal structure and function of adult hearts through production of ECM, which is composed of collagen, laminin, fibronectin, fibrillin, elastin, proteoglycan, and other components (Lockhart et al., 2011; Hortells et al., 2019; Souders et al., 2009). ECM proteins not only provide mechanical support for other cell lineages, but also determine the biomechanical characteristics such as stiffness of cardiac tissue, thus generating a microenvironment for cardiomyocyte proliferation.

Although generally in a low proliferative state, cardiac fibroblasts are activated through cytokine stimulation upon injury (Stempien-Otero et al., 2016). A subpopulation of these activated fibroblasts further differentiate into myofibroblasts, initially defined by the expression of alpha-smooth muscle actin (α-SMA) (Souders et al., 2009; Snider et al., 2009). Recent studies have identified Periostin as another marker that is only expressed after injury and labels nearly all myofibroblasts (Snider et al., 2009; Kaur et al., 2016; Kanisicak et al., 2016). These activated fibroblasts present pro-angiogenic and pro-fibrotic activities, which are important for collagen formation and subsequent cardiac tissue wound healing. Thus, elimination of those cells often results in ventricular rupture (Kanisicak et al., 2016). In addition, cardiac ECM generated by fibroblasts also plays an essential role during regeneration. As previously mentioned, heparan sulfate proteoglycan agrin has been identified as critical for neonatal mouse heart regeneration through the disassembly of DGC. In infarcted adult mouse heart, a single administration of agrin promotes cardiac function recovery (Bassat et al., 2017). Furthermore, decreasing ECM stiffness is able to prolong the time window of regenerative capacity in neonatal mouse heart (Notari et al., 2018). However, in lower vertebrates including zebrafish (Sanchez-Iranzo et al., 2018), newt (Mercer et al., 2013) and axolotl (Godwin et al., 2017), although studies have documented the potential involvement of fibroblasts during heart regeneration, their specific functions remain largely unexplored, partly results from a lack of specific genetic markers to identify fibroblasts.

Despite this beneficial role for cardiac repair during the acute phase, activation of fibroblasts leads to fibrosis, which can contribute to impaired cardiac function and arrhythmias over the long term. Consequently, direct reprogramming of fibroblasts to cardiomyocytes could have beneficial effects on injured heart, given the abundant pool of fibroblasts within the heart. A recent study has utilized a retroviral system to deliver a set of core transcription factors including Gata4, Hand2, Mef2c, and Tbx5 (GHMT) into injured hearts. After 4 weeks, fibroblasts that have been labelled by lineage-tracing strategy could be successfully reprogrammed into cardiac-like myocytes in vivo (Song et al., 2012). Likewise, another study has shown similar results by utilizing Gata4, Mef2c, and Tbx5 (GMT) combination (Qian et al., 2012). In both conditions, pronounced functional improvement of cardiac function has been observed. However, it is worth noting that in another study, GMT overexpression in fibroblasts is inefficient to induce the electrophysiological characteristics of mature cardiomyocytes (Chen et al., 2012). Such discrepancy might result from the utilization of different protocols or mouse strains, or the inherent heterogeneity of cardiac fibroblasts. To this end, single-cell transcriptome study has been used to study the temporal dynamics of gene expression in different subpopulations of fibroblasts during reprogramming, which has further identified Ptbp1 as a critical barrier for reprogramming efficiency (Liu et al., 2017). In addition, these directly reprogrammed cardiomyocytes in vivo are more mature and closely resemble endogenous cardiomyocytes, compared with in vitro reprogramming using similar methods (Song et al., 2012; Qian et al., 2012). This observation might be related to the native microenvironment of the intact heart, including locally secreted growth factors, distinct tissue stiffness, and contractile properties with the presence of ECM.

#### Immune cells

Cardiac injury is accompanied by activation of immune response and robust infiltration of immune cells, which are essential for both acute cardiac wound healing and heart regeneration. We focus on the different functions of two types of immune cells, macrophages and regulatory T-cells (Tregs), which mediate innate and adaptive immune response, respectively. **Macrophages**: During heart development, macrophages facilitate cardiac electrical conduction (Hulsmans et al., 2017) and promote coronary vasculature formation (Leid et al., 2016). After cardiac injury, innate immunity mediated by tissue resident macrophages promotes angiogenesis and ECM remodeling to enhance regeneration (Vannella & Wynn, 2017; de Couto, 2019). In zebrafish (Lai et al., 2017), axolotl (Godwin et al., 2017) or mouse (Aurora et al., 2014) models, clodronate liposomes mediated macrophage depletion consistently leads to compromised neovascularization and cardiomyocytes proliferation, suggesting a conserved role of macrophages in regeneration across species. Interestingly, medaka, another lower vertebrate teleost which share similar cardiac structure and living environment with zebrafish, fail to regenerate injured hearts due to delayed and reduced macrophage recruitment. Accordingly, stimulating Toll-like receptor signaling in medaka promotes heart regeneration (Lai et al., 2017). Furthermore, in mammalian hearts, tissue resident macrophages are not homogenous and can be further divided into two subpopulations based on the expression of C-C chemokine receptor 2 (CCR2) (Epelman et al., 2014a; Epelman et al., 2014b). CCR2^−^ macrophages are derived from embryonic progenitors and seed the heart during early fetal and perinatal stages. In contrast, CCR2^+^ macrophages are derived from definitive hematopoietic stem cells and are replaced slowly by circulating monocytes (Epelman et al., 2014a). Recent study further demonstrates CCR2^−^ and CCR2^+^ macrophages play opposite roles in monocyte recruitment after cardiac injury, and selectively depletion of these two subsets results in divergent effects on heart remodeling (Bajpai et al., 2019). In addition, the role for macrophages during heart regeneration has been studied in the context of stem cell therapy. Regional CCR2^+^ and C-X3-C motif chemokine receptor 1 (CX3CR1^+^) macrophages accumulation alters fibroblast activity, decreases ECM in wound border zone, as well as improves mechanical properties of the heart (Vagnozzi et al., 2020). **Tregs**: As a key adaptive immune response mediator, Tregs can directly promote zebrafish or neonatal mouse heart regeneration. In zebrafish, *forkhead box P3a*^*+*^ (*foxp3a*^+^) Tregs stimulate regeneration by producing Nrg1 to enhance cardiomyocytes proliferation. Consequently, Tregs depleted hearts display regeneration defects with deposition of fibrin and formation of collagenous scar (Hui et al., 2017). In neonatal mouse, CD4^+^ Tregs depletion through genetic ablation or the lytic anti-CD25 antibody treatment results in reduced heart regeneration (Li et al., 2019; Fung et al., 2020; Li et al., 2020). Moreover, Tregs deficient NOD/SCID mouse display regeneration defects, which can be rescued by adoptive transfer of Tregs (Li et al., 2019). Mechanistically, Tregs potentiate neonatal cardiomyocyte proliferation through secreted factors such as chemokine ligand 24 (CCL24), growth arrest specific 6 (GAS6) and amphiregulin (AREG) (Li et al., 2019).

### Concluding remarks and outlook

The utilization of various new genetic tools in the past two decades has significantly advanced our understanding of heart regeneration, especially on zebrafish and mouse models. From acute wound healing responses to long term cardiomyocyte proliferation, spatiotemporal activation of multiple signaling pathways in different cell types are required to reconstruct an injured heart. However, there are many important questions that remain to be answered. What is the molecular mechanism that initiates the cell cycle exit of mammalian cardiomyocytes after birth? Which molecule triggers the initial regenerative responses? How to induce reprogramming and cell division of mature mammalian cardiomyocytes in vivo? The answers to these or other related questions will help us to understand the molecular and cellular mechanisms underlying heart regeneration in model organisms, and more importantly, will set the stage for the development of strategies to either promote the intrinsic proliferative potential of cardiomyocytes, or optimize exogenous stem cell based methods for cardiac diseases therapy.
